# Correction: Rapelli et al. Recognizing and Appreciating the Partner’s Support Protects Relationship Satisfaction during Cardiac Illness. *J. Clin. Med.* 2024, *13*, 1180

**DOI:** 10.3390/jcm13237473

**Published:** 2024-12-09

**Authors:** Giada Rapelli, Silvia Donato, Emanuele Maria Giusti, Giada Pietrabissa, Miriam Parise, Ariela Francesca Pagani, Chiara A. M. Spatola, Anna Bertoni, Gianluca Castelnuovo

**Affiliations:** 1 Department of Medicine and Surgery, University of Parma, 43125 Parma, Italy; giada.rapelli@unipr.it; 2 Department of Psychology, Catholic University of Sacred Heart, 20123 Milan, Italy; silvia.donato@unicatt.it (S.D.); miriam.parise@unicatt.it (M.P.); anna.bertoni@unicatt.it (A.B.); gianluca.castelnuovo@auxologico.it (G.C.); 3 Family Studies and Research University Centre, Catholic University of Sacred Heart, 20123 Milan, Italy; 4 EPIMED Research Center, Department of Medicine and Surgery, University of Insubria, 21100 Varese, Italy; emanuelemaria.giusti@uninsubria.it; 5 Clinical Psychology Research Laboratory, IRCCS Istituto Auxologico Italiano, 20149 Milan, Italy; 6 Department of Humanities, University of Urbino Carlo Bo, 61029 Urbino, Italy; ariela.pagani@uniurb.it; 7 Department of Clinical and Experimental Medicine, University of Messina, 98122 Messina, Italy; chiara.spatola@unime.it

In the published publication [1], there was an error regarding the affiliations for Giada Pietrabissa and Gianluca Castelnuovo. In addition to affiliation 2, the updated affiliation should include the following: “5. Clinical Psychology Research Laboratory, IRCCS Istituto Auxologico Italiano, 20149 Milan, Italy”. The authors state that the scientific conclusions are unaffected. This correction was approved by the Academic Editor. The original publication has also been updated.

## References

[1] Rapelli G., Donato S., Giusti E.M., Pietrabissa G., Parise M., Pagani A.F., Spatola C.A.M., Bertoni A., Castelnuovo G. (2024). Recognizing and Appreciating the Partner’s Support Protects Relationship Satisfaction during Cardiac Illness. J. Clin. Med..

